# Supplementary material for: A novel twelve class fluctuation test reveals higher than expected mutation rates for influenza A viruses
Source: eLife. 2017 Jun 9;6:e26437. doi: 10.7554/eLife.26437 (PMC5511008; doi:10.7554/eLife.26437)
Supplement: Supplementary file 1. — DOI: http://dx.doi.org/10.7554/eLife.26437.017 [file elife-26437-supp1.docx]

**Supplementary File 1.** Nonsense mutation counts from PrimerID sequencing of the influenza PA gene.

|  | A->T | C->A | C->G | C->U | G->A | G->U | U->A | U->G |
| --- | --- | --- | --- | --- | --- | --- | --- | --- |
| Nonsense mutation targets per 402 base consensus sequence | 16 | 9 | 7 | 5 | 10 | 19 | 9 | 5 |
| Nonsense mutations found in 449,655 cell derived RNA consensus sequences | 5 | 10 | 33 | 9 | 583 | 35 | 16 | 1 |
| Nonsense mutations found in 481,286 viral derived RNA consensus sequences | 10 | 31 | 22 | 11 | 407 | 30 | 15 | 3 |
