# Supplementary material for: A novel twelve class fluctuation test reveals higher than expected mutation rates for influenza A viruses
Source: eLife. 2017 Jun 9;6:e26437. doi: 10.7554/eLife.26437 (PMC5511008; doi:10.7554/eLife.26437)
Supplement: Supplementary file 2. — DOI: http://dx.doi.org/10.7554/eLife.26437.018 [file elife-26437-supp2.docx]

**Supplementary File 2**: Influenza A virus mutation rates

|  |  | |
| --- | --- | --- |
|  | Mutation rate (mutations per nt per strand replicated)^a^ | |
| Mutation class | A/Puerto Rico/8/1934 H1N1 | A/Hong Kong/4801/2014 H3N2 |
| A to C | 1.5 x 10^-5^ ± 0.9 x 10^-5^ | 3.4 x 10^-5^ ± 1.0 x 10^-5^ |
| A to G | 2.0 x 10^-4^ ± 1.1 x 10^-4^ | 3.0 x 10^-4^ ± 1.5 x 10^-4^ |
| A to U | 1.8 x 10^-5^ ± 1.8 x 10^-5^ | 1.3 x 10^-5^ ± 0.3 x 10^-5^ |
| C to A | 7.7 x 10^-6^ ± 4.4 x 10^-6^ | 1.7 x 10^-5^ ± 0.9 x 10^-5^ |
| C to G | 5.1 x 10^-6^ ± 2.3 x 10^-6^ | 9.7 x 10^-6^ ± 7.8 x 10^-6^ |
| C to U | 2.7 x 10^-5^ ± 0.7 x 10^-5^ | 4.6 x 10^-5^ ± 1.6 x 10^-5^ |
| G to A | 3.1 x 10^-5^ ± 0.2 x 10^-5^ | 7.2 x 10^-5^ ± 1.1 x 10^-5^ |
| G to C | 5.4 x 10^-5^ ± 2.4 x 10^-5^ | 2.8 x 10^-5^ ± 0.7 x 10^-5^ |
| G to U | 3.5 x 10^-5^ ± 0.9 x 10^-5^ | 6.0 x 10^-5^ ± 1.6 x 10^-5^ |
| U to A | 1.4 x 10^-5^ ± 0.7 x 10^-5^ | 4.5 x 10^-6^ ± 1.8 x 10^-6^ |
| U to C | 2.3 x 10^-4^ ± 0.5 x 10^-4^ | 3.1 x 10^-4^ ± 1.2 x 10^-4^ |
| U to G | 3.5 x 10^-5^ ± 2.3 x 10^-5^ | 3.6 x 10^-5^ ± 2.3 x 10^-5^ |
| Overall^b^ | 1.8 x 10^-4^ | 2.5 x 10^-4^ |

^a^ Arithmetic mean plus or minus the standard deviation calculated from at least three replicates.

^b^ Overall mutation rates were determined as a weighted average of mutation rates, taking into account genomic base composition.
